# A Fatal Case of Chronic Granulomatous Disease in a Young Man

**DOI:** 10.7759/cureus.40266

**Published:** 2023-06-11

**Authors:** Ngoc Huong Helen Ho, Siddharth Patel, Prutha Pathak

**Affiliations:** 1 Internal Medicine, Alabama College of Osteopathic Medicine, Dothan, USA; 2 Internal Medicine, Decatur Morgan Hospital, Decatur, USA; 3 Maternal and Child Health, Gillings School of Global Public Health, Chapel Hill, USA

**Keywords:** rare autoimmune disease, antimicrobial prophylaxis, catalase positive, nadph oxidase, chronic granulomatous disease

## Abstract

Chronic granulomatous disease (CGD) is a rare X-linked or autosomal recessive disorder of early childhood due to defective nicotinamide adenine dinucleotide phosphate (NADPH) oxidase enzyme in leukocytes. It increases susceptibility to infections by catalase-positive bacteria and fungi. We report a case of an 18-year-old man with CGD who presented to the hospital with septic shock due to bacteremia, pneumonia, and osteomyelitis due to multiple rare microorganisms. Despite aggressive management, he did not survive. Increasing awareness about the common infections in this rare disease, their prevention, and lifelong treatment is warranted.

## Introduction

Chronic granulomatous disease (CGD) is a rare X-linked or autosomal recessive disorder that usually presents in early childhood between one to three years of age [1]. It affects about one in 250,000 individuals annually due to a defect in the nicotinamide adenine dinucleotide phosphate (NADPH) oxidase enzyme in phagocytes [2]. The defect decreases the production of reactive oxygen species (ROS) for the respiratory burst reaction required to eliminate microorganisms. Individuals with CGD are prone to recurrent infections due to catalase-positive organisms such as Staphylococcus, Burkholderia, Aspergillus, Serratia, and Norcardia [3]. Early detection and treatment of infections and life-long antibiotic and antifungal prophylaxis are essential for survival. Unfortunately, many patients with CGD die in childhood due to infectious complications and survival up to adulthood has not been common until recently. We report a case of an 18-year-old man with a history of CGD and pulmonary aspergillosis who presented with recurrent catalase-positive infections.

## Case presentation

An 18-year-old man presented to the Emergency Room (ER) with shortness of breath and cough of three weeks duration. He also reported low-grade fever and episodic vomiting. His past medical history was significant for CGD since early childhood, and a three-year history of pulmonary aspergillosis. Unfortunately, he was non-compliant with prophylactic levofloxacin and voriconazole. The patient’s family history was significant for CGD in his brother.

**Figure 1:**
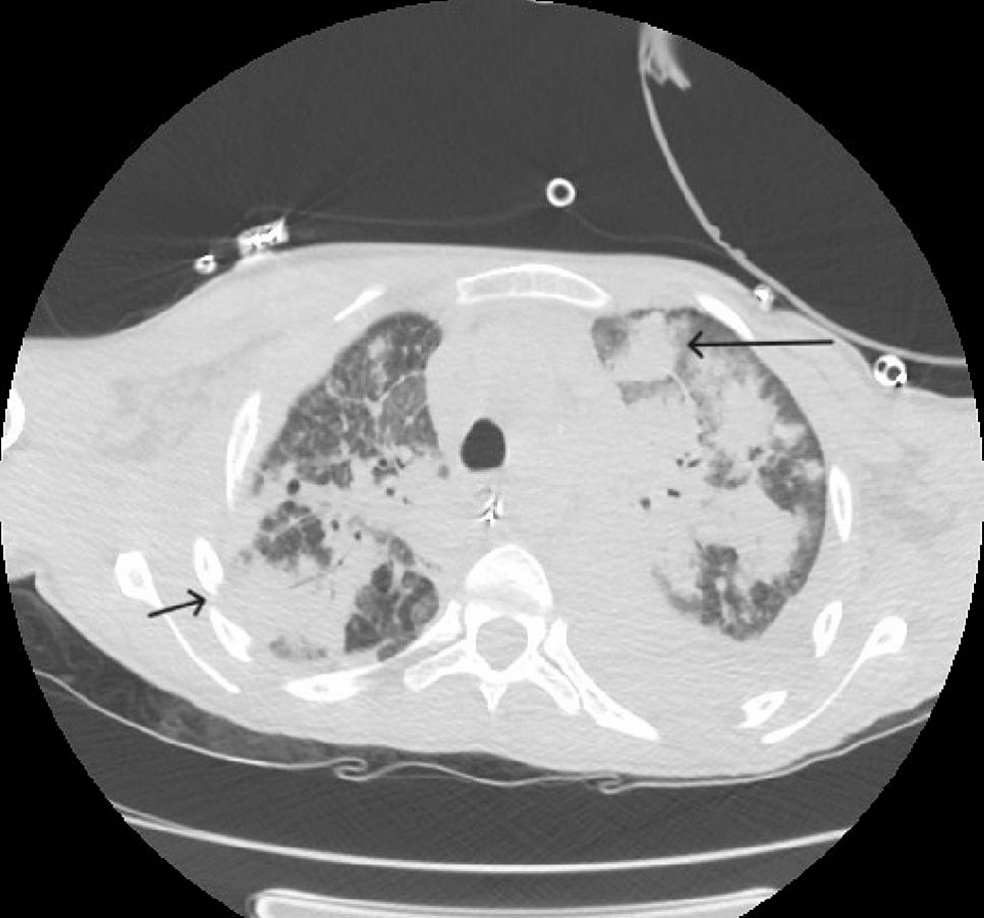
CT chest with IV contrast demonstrating pulmonary nodules surrounded by ground glass opacities (big arrow) and bony destruction of ribs (small arrow) CT: Computed Tomography, IV: Intravenous

**Figure 2:**
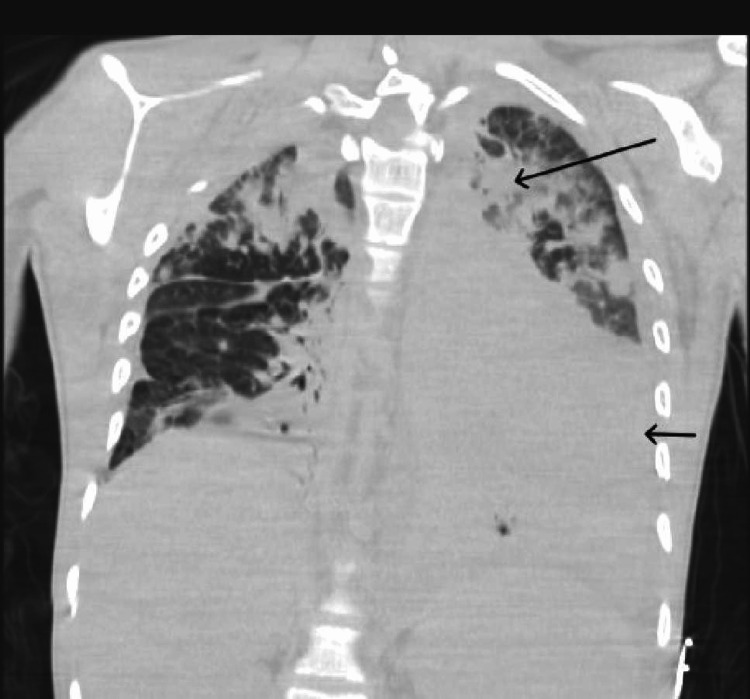
CT chest with IV contrast demonstrating a large left pleural effusion (small arrow) and pulmonary nodules surrounded by ground glass opacities (large arrow) CT: Computed Tomography, IV: Intravenous

On physical examination, he had a temperature of 97.6 F, blood pressure of 97/61 mmHg, pulse of 137 per minute, respiratory rate of 30 per minute, and oxygen saturation of 82% on room air. He was in respiratory distress with diffuse bilateral crackles on lung examination. Despite initial resuscitation with fluids and oxygen, his hemodynamic instability escalated to require emergent intubation, initiation of mechanical ventilation, and vasopressors within a short time after the presentation. Laboratory results revealed leukocytosis, elevated transaminases, and acute renal failure (Table 1). Chest imaging showed bilateral nodular alveolar opacities, left pleural effusion, and bony destruction of the ribs (Figures 1, 2). Intravenous vancomycin, meropenem, and voriconazole were started, and he was admitted to the intensive care unit for further care.

**Table 1:** Blood investigations on admission

Test	Result	Reference range
White Blood Cell count	130300 cells/mm^3^	4.8-10.8 x 1000
Hemoglobin	11.7 g/dL	14.0-18.0 g/dL
Platelet	218 x 1000 cells/mm^3^	130-400 x 1000 cells/mm^3^
Sodium	138 mmol/L	136-145 mmol/L
Potassium	5.6 mmol/L	3.5-5.1 mmol/L
Chloride	104 mmol/L	98-107 mmol/L
Carbon Dioxide	20 mmol/L	25-35 mmol/L
Anion Gap	14	8-14
Blood Urea Nitrogen	25 mg/dL	8-22 mg/dL
Glucose	158 mg/dL	70-104 mg/dL
Creatinine	1.0 mg/dL	0.7-1.2 mg/dL
Calcium	8.2 mg/dL	8.8-10.2 mg/dL
Ferritin	7717 ng/dL	6-320 ng/dL
Aspartate Aminotransferase	203 U/L	10-34 U/L
Alanine Aminotransferase	155 U/L	10-44 U/L
Alkaline Phosphatase	431 U/L	30-224 U/L
Lactate Dehydrogenase	429 U/L	135-225 U/L
Creatine Kinase	22 U/L	24-204 U/L
C-Reactive Protein, Quantitative	252.84 mg/L	0-5 mg/L
Pro-B-Natriuretic Peptide	2947 pg/mL	5-93 pg/mL
Total Protein	5.9 g/dL	6.3-8.3 g/dL
Albumin	2.4 g/dL	3.5-5.0 g/dL
Lipase	7 U/L	13-60 U/L
Aspergillus galactomannan Antigen	>=3.750	<0.5 index
Beta-(1,3)-D-Glucan	339 pg/mL	<60 pg/mL
Blood culture	Burkholderia Cepacia, Staphylococcus Hominis	

Screening for Cryptococcus, Histoplasma, Legionella, Streptococcus, and SARS were negative. Screening for Aspergillus and Beta-(1, 3)-D-Glucan were positive (Table 1). Blood cultures returned positive for Burkholderia cepacia and Staphylococcus hominis. As a result, diagnoses of septic shock due to bilateral multifocal pneumonia, aspergillus osteomyelitis, and polymicrobial bacteremia were established. Unfortunately, the patient’s condition deteriorated over the next 48 hours with worsening hypoxia, hypotension, and multi-organ dysfunction. Although renal replacement therapy was attempted, it was interrupted due to hemodynamic instability. Ultimately, he went into cardiac arrest, and despite aggressive measures, recovery was unsuccessful, and the patient expired.

## Discussion

CGD is an inherited immunodeficiency disorder due to a defective NADPH oxidase enzyme in phagocytes, which presents with recurrent infections and a persistent inflammatory state [4]. Genetic mutations in the structural proteins of NADPH oxidase complex lead to decreased hydrogen peroxide and ROS production, and inadequate respiratory burst [1,2,4]. Catalase-positive microorganisms break down even the limited hydrogen peroxide in phagocytes, making those with CGD more vulnerable to these species. Aspergillus, Staphylococcus aureus, Nocardia, Burkholderia, and Serratia are the most common organisms causing pneumonia, bacteremia, osteomyelitis, cellulitis, and abscess formation in CGD patients [5]. Additional complications arise from increased inflammatory response due to poor clearance of microbial material [3,4]. Phagocyte accumulation forms non-caseating granulomas, especially in the lungs and gastrointestinal tract [3,4]. Patients may also develop hepatosplenomegaly, chorioretinitis, interstitial pulmonary fibrosis, and inflammatory bowel disease [6].

Dihydrorhodamine 123 test (DHR) or Nitroblue Tetrazolium (NBT) test is performed to diagnose CGD. Following positive results, genetic testing is performed to determine the type of CGD: X-linked or autosomal recessive [7]. Lifelong prophylaxis with an antibiotic, e.g., trimethoprim-sulfamethoxazole, and an antifungal, e.g., itraconazole, remains a cornerstone in managing CGD [8]. However, infections occur in about 0.3% of patients with CGD per year, regardless [4]. Prompt treatment of acute infections with empiric antimicrobials targeting the above organisms is crucial [2,4]. Additional treatment with interferon-gamma (IFN-y) and corticosteroids is being considered but large-scale clinical trials have not studied their efficacy [4]. Although a cure for CGD is still being investigated, advanced knowledge in its management has recently improved median life expectancy to around 30-40 years. Hematopoietic stem cell transplantation and gene therapy are treatment alternatives but accompany their own risks [9,10].

The patient in the present case was non-compliant with the antimicrobial prophylaxis for about one year despite having a past history of pulmonary aspergillosis. This drastically placed him at extreme risk for recurrent infections. Also, there was a significant delay in seeking medical attention. Despite aggressive resuscitation and the institution of broad-spectrum antibiotics and antifungals, an untoward outcome could not be averted.

## Conclusions

This case provides a classic clinical presentation of a rare clinical entity. The patient was found to have infections with two catalase-positive organisms: Burkholderia and Aspergillus. His imaging also revealed bilateral diffuse granulomas classically seen in pulmonary aspergillosis. Pneumonia and osteomyelitis due to such rare organisms leading to septic shock were unique to this case. Although rare, this case highlights the importance of knowledge of the infections CGD patients are susceptible to. Increasing life expectancy among these patients means more patients with CGD present with infectious manifestations in adulthood. Hence, knowledge about CGD, early diagnosis, and aggressive management of infections are essential. Life-long prophylaxis with antimicrobials reduces the rate of severe infections and remains the cornerstone of CGD management.
